# On new sixth and seventh order iterative methods for solving non-linear equations using homotopy perturbation technique

**DOI:** 10.1186/s13104-022-06154-5

**Published:** 2022-07-30

**Authors:** Srinivasarao Thota, P. Shanmugasundaram

**Affiliations:** 1 Department of Mathematics, School of Sciences, SR University, Warangal, Telangana 506371 India; 2 Department of Mathematics, College of Natural & Computational sciences, Mizan Tepi University, Mizan Teferi, Ethiopia

**Keywords:** Iterative methods, Nonlinear equations, Order of convergence, Homotopy perturbation technique, 65N30, 49M37

## Abstract

**Objectives:**

This paper proposes three iterative methods of order three, six and seven respectively for solving non-linear equations using the modified homotopy perturbation technique coupled with system of equations. This paper also discusses the analysis of convergence of the proposed iterative methods.

**Results:**

Several numerical examples are presented to illustrate and validation of the proposed methods. Implementation of the proposed methods in Maple is discussed with sample computations.

## Introduction

The applications of non-linear equations of the type $f(x)=0$ arise in various branches of pure and applied sciences, engineering and computing. In resent time, several scientists and engineers have been focused to solve non-linear equations numerically as well as analytically. In the literature, there are several iterative methods/algorithms available which are derived from different techniques such as homotopy, interpolation, Taylor’s series, quadrature formulas, decomposition etc., and also available various modifications and improvements of the existing methods, and different hybrid iterative methods, see, for example [1, 4–7, 9–16, 28–32, 36–38]. In general, the roots of non-linear or transcendental equations cannot be expressed in closed form or cannot be computed analytically. The root-finding algorithms provide us to compute approximations to the roots, these approximations are expressed either as small isolating intervals or as floating point numbers. In this paper, we use the modified homotopy perturbation technique (HPT) to create a number of iterative methods for solving the given non-linear equations with converging order more than or equal to three. The given non-linear equations are expressed as an equivalent coupled system of equations with help of the Taylor’s series and technique of He [4]. This enables us to express the given non-linear equation as a sum of linear and non-linear equations. The Maple implementation of the proposed algorithm is also discussed, and various Maple implementations for differential and transcendental equations are available in the literature, see, for example [17–27].

The rest of paper is organized as follows: Section  recalls the preliminary concepts related to the topic; In Section , we present the methodology and steps involving in the proposed algorithms; Section  discuses the analysis of convergence to show the order of proposed methods are more than or equal to three; Section  presents several numerical examples to illustrate and validate the proposed methods/algorithms; and finally Section  presents the Maple implementation of the proposed algorithms with sample computations.

### Preliminaries

In this paper, we consider the non-linear equation of the type

1 $$\begin{aligned} f(x) = 0. \end{aligned}$$

Iterations techniques are a common approach widely used in various numerical algorithms/methods. It is a hope that an iteration in the general form of $x_{n+1}=g(x_n)$ will eventually converge to the true solution $\alpha$ of the problem (1) at the limit when $n\rightarrow \infty$. The concern is whether this iteration will converge, and, if so, the rate of convergence. Specifically we use the following expression to represent how quickly the error $e_n=\alpha -x_n$ converges to zero. Let $e_n=\alpha -x_n$ and $e_{n+1} = \alpha -x_{n+1}$ for $n \ge 0$ be the errors at *n*-th and $(n+1)$-th iterations respectively. If two positive constants $\mu$ and *p* exist, and

2 $$\begin{aligned} \lim \limits _{n\rightarrow \infty }\frac{\vert e_{n+1}\vert }{\vert e_n \vert ^p}=\frac{\vert \alpha -x_{n+1}\vert }{\vert \alpha -x_n\vert ^p}=\mu , \end{aligned}$$

then the sequence is said to converge to $\alpha$. Here $p\ge 1$ is called the *order of convergence*, the constant $\mu$ is the *rate of convergence* or *asymptotic error constant*. This expression may be better understood when it is interpreted as $\vert e_{n+1}\vert =\mu \vert e_n\vert ^p$ when $n\rightarrow \infty$. Obviously, the larger *p* and the smaller $\mu$, the more quickly the sequence converges.

#### Theorem 1

[3] Suppose that $\phi \in C^p[a,b]$. If $\phi ^{(k)}(x)=0$, for $k=0, 1, 2, \ldots , p-1$ and $\phi ^{(p)}(x) \ne 0$, then the sequence $\{x_n\}$ is of order *p*.

This paper focus on developing iterative methods/algorithms that are having the order of converges three, six and seven respectively. The following section presents the proposed methods using Taylor’s series and modified HPT.

## Main text

In this section, we present new iterative methods and its order of convergences with numerical examples, maple implementation and sample computations using maple mathematical software tool.

### New iterative methods

We assume that $\alpha$ is an exact root of the equation (1) and let *a* be an initial approximation (sufficiently close) to $\alpha$. We can rewrite the non-linear equation (1) using Taylor’s series expansion as coupled system

3 $$\begin{aligned} f(x)= f(a) + (x-a)f'(a) + \frac{(x-a)^2}{2} f''(a) + G(x) = 0~~\text {or} \end{aligned}$$

4 $$\begin{aligned} G(x)= f(x) - f(a) - (x-a)f'(a) - \frac{(x-a)^2}{2} f''(a). \end{aligned}$$

We have, from Newton’s method, that

5 $$\begin{aligned} {\begin{matrix}x=a-\frac{f(a)}{f'(a)} \implies x-a = -\frac{f(a)}{f'(a)} \implies (x-a)^2 = \left( -\frac{f(a)}{f'(a)}\right) ^2. \end{matrix}} \end{aligned}$$

From (4) and (5), we have

6 $$\begin{aligned} G(x) = f(x) - f(a) - (x-a)f'(a) - \frac{1}{2} \left( -\frac{f(a)}{f'(a)}\right) ^2 f''(a). \end{aligned}$$

We can write (6) in the following form

$$\begin{aligned} x = a- \frac{f(a)}{f'(a)}-\frac{(f(a))^2f''(a)}{2(f'(a))^3} - \frac{G(x)}{f'(a)}. \end{aligned}$$

It can be expressed in the form of

7 $$\begin{aligned} x=c+T(x), \end{aligned}$$

where

8 $$\begin{aligned}c = a- \frac{f(a)}{f'(a)}-\frac{(f(a))^2f''(a)}{2(f'(a))^3}, \end{aligned}$$

9 $$\begin{aligned}T(x) = - \frac{G(x)}{f'(a)}. \end{aligned}$$

Here *T*(*x*) is a non-linear operator. It is clear, from relation (4), that

10 $$\begin{aligned} G(x_0) = f(x_0). \end{aligned}$$

Note that the equation (10) will play important role in the derivation of the iteration methods, see for example [2]. We use the technique of homotopy perturbation to develop the proposed iterative algorithms to solve the given non-linear equation (1). Using the HPT, we can construct a homotopy $H(\upsilon ,p,m) : \mathbb {R} \times [0,1] \times \mathbb {R} \rightarrow \mathbb {R}$ satisfying

11 $$\begin{aligned} H(\upsilon ,p,m) = \upsilon -c-pT(\upsilon ) - p(1-p)m=0, \end{aligned}$$

where $p\in [0,1]$ is embedding parameter and $m \in \mathbb {R}$ is unknown number. Clearly, from (11), we have

$$\begin{aligned}H(\upsilon ,0,m) = \upsilon -c=0, ~~\text {and}\\H(\upsilon ,1,m) = \upsilon -c-T(\upsilon )=0. \end{aligned}$$

Hence, the parameter *p* is monotonically increases on [0, 1]. The solution of equation (11) can be expressed as a power series in *p*

12 $$\begin{aligned} \upsilon = \sum _{i=0}^{\infty } v_ip^i. \end{aligned}$$

Now the approximate solution of (1) is

13 $$\begin{aligned} x=\lim \limits _{p \rightarrow 1} \upsilon = \sum _{i=0}^{\infty } x_i. \end{aligned}$$

One can express the equation (11), as follows, by expanding *T*(*x*) using Taylor’s series expansion around $x_0$,

14 $$\begin{aligned} \upsilon -c-p \left[ T(x_0) + (\upsilon -x_0)T'(x_0) + \frac{(\upsilon -x_0)^2}{2} T''(x_0) + \cdots \right] - p(1-p)m=0. \end{aligned}$$

By Putting (12) in (14), we get

15 $$\begin{aligned} {\begin{matrix} {} \sum _{i=0}^{\infty } v_ip^i-c - p(1-p)m \\ {}\quad - p \left[ T(x_0) + \left( \sum _{i=0}^{\infty } v_ip^i-x_0 \right) T'(x_0) + \left( \sum _{i=0}^{\infty } v_ip^i-x_0 \right) ^2 \frac{T''(x_0)}{2} + \cdots \right] = 0. \end{matrix}} \end{aligned}$$

By comparing the coefficients of powers of *p*, we get

16 $$\begin{aligned}p^0: x_0 - c=0 \end{aligned}$$

17 $$\begin{aligned}P^1: x_1 - T(x_0) - m =0 \end{aligned}$$

18 $$\begin{aligned}p^2: x_2 - x_1T'(x_0) + m = 0 \end{aligned}$$

19 $$\begin{aligned}p^3: x_3 - x_2T'(x_0) - \frac{1}{2} x_1^2T''(x_0)=0 \nonumber \\\quad \vdots \end{aligned}$$

From (17), we have $x_1 = T(x_0) + m$. To obtain the value of *m*, assume $x_2=0$. Now from (18)

20 $$\begin{aligned} m = \frac{T(x_0)T'(x_0)}{1-T(x_0)}. \end{aligned}$$

Now, $x_0,x_1,x_2,x_3,\ldots$ are obtained as follows. From (16), we have

21 $$\begin{aligned} x_0 = c \implies x_0 = a- \frac{f(a)}{f'(a)}-\frac{(f(a))^2f''(a)}{2(f'(a))^3}. \end{aligned}$$

From (17) and (20), we have

22 $$\begin{aligned} x_1 = T(x_0) + m = \frac{T(x_0)}{1-T'(x_0)}. \end{aligned}$$

From the assumption $x_2=0$ and from (19), we get

23 $$\begin{aligned} x_3 = x_2T'(x_0) + \frac{1}{2} x_1^2T''(x_0) = \frac{T^2(x_0)T''(x_0)}{2(1-T'(x_0))^2}. \end{aligned}$$

From (6), (10) and (9), we have

24 $$\begin{aligned}T(x_0) = - \frac{G(x_0)}{f'(a)} = -\frac{f(x_0)}{f'(a)}, \end{aligned}$$

25 $$\begin{aligned}T'(x_0) = -\frac{G'(x_0)}{f'(a)} = -\frac{f'(x_0)-f'(a)}{f'(a)} = 1-\frac{f'(x_0)}{f'(a)}, \end{aligned}$$

26 $$\begin{aligned}T''(x_0) = -\frac{G''(x_0)}{f'(a)} = -\frac{f''(x_0)}{f'(a)}. \end{aligned}$$

The approximate solution is obtained as

27 $$\begin{aligned} x = \lim \limits _{i\rightarrow \infty } x_i = x_0 + x_1 + x_2 + \cdots + x_i. \end{aligned}$$

This formulation allows us to form the following iterative methods.

#### Algorithm 1

For $i=0$, we have

$$\begin{aligned} x \approx x_0 = a- \frac{f(a)}{f'(a)}-\frac{(f(a))^2f''(a)}{2(f'(a))^3}. \end{aligned}$$

Hence, for a given $x_0$, we have the following iterative formula to find the approximate solution $x_{n+1}$.

28 $$\begin{aligned} x_{n+1} = x_n- \frac{f(x_n)}{f'(x_n)}-\frac{(f(x_n))^2f''(x_n)}{2(f'(x_n))^3}. \end{aligned}$$

#### Algorithm 2

For $i=1$, we have

$$\begin{aligned} x \approx x_0 + x_1= a - \frac{f(a)}{f'(a)}-\frac{(f(a))^2f''(a)}{2(f'(a))^3} + \frac{T(x_0)}{1-T'(x_0)} \\~ = a - \frac{f(a)}{f'(a)}-\frac{(f(a))^2f''(a)}{2(f'(a))^3} - \frac{f(x_0)}{f'(x_0)} \end{aligned}$$

Hence, for a given $x_0$, we have the following iterative schemes to find the approximate solution $x_{n+1}$.

29 $$\begin{aligned} {\begin{matrix} y_n {}= x_n- \frac{f(x_n)}{f'(x_n)}-\frac{(f(x_n))^2f''(x_n)}{2(f'(x_n))^3}, \\ x_{n+1} {}= x_n - \frac{f(x_n)}{f'(x_n)}-\frac{(f(x_n))^2f''(x_n)}{2(f'(x_n))^3} - \frac{f(y_n)}{f'(y_n)}. \end{matrix}} \end{aligned}$$

**Note:** Since $x_2=0$, we have the formula (29) for $i=2$. i.e., $x \approx x_0 + x_1 = x_0 + x_1 + x_2$.

#### Algorithm 3

For $i=3$, we have

$$\begin{aligned} x \approx~ x_0 + x_1 + x_2 + x_3 \\~ = a - \frac{f(a)}{f'(a)}-\frac{(f(a))^2f''(a)}{2(f'(a))^3} + \frac{T(x_0)}{1-T'(x_0)} + \frac{T^2(x_0)T''(x_0)}{2(1-T'(x_0))^2}\\~ = a - \frac{f(a)}{f'(a)}-\frac{(f(a))^2f''(a)}{2(f'(a))^3} - \frac{f(x_0)}{f'(x_0)} - \frac{(f(x_0))^2f''(x_0)}{2f'(a)(f'(x_0))^2} \end{aligned}$$

Hence, for a given $x_0$, we have the following iterative formula to find the approximate solution $x_{n+1}$.

30 $$\begin{aligned} {\begin{matrix} y_n {}= x_n- \frac{f(x_n)}{f'(x_n)}-\frac{(f(x_n))^2f''(x_n)}{2(f'(x_n))^3}, \\ x_{n+1} {}= x_n - \frac{f(x_n)}{f'(x_n)}-\frac{(f(x_n))^2f''(x_n)}{2(f'(x_n))^3} - \frac{f(y_n)}{f'(y_n)} - \frac{(f(y_n))^2f''(y_n)}{2f'(x_n)(f'(y_n))^2}. \end{matrix}} \end{aligned}$$

### Order of convergence

In this section, we show, in the following theorems, that the orders of converges of Algorithms 1, 2 and 3 are three, six and seven respectively. Let $I \subset \mathbb {R}$ be an open interval. To prove this, we follow the proofs of [9, Theorem 5, Theorem 6].

#### Theorem 2

Let $f:I \rightarrow \mathbb {R}$. Suppose $\alpha \in I$ is a simple root of (1) and $\theta$ is a sufficiently small neighborhood of $\alpha$. Let $f''(x)$ exist and $f'(x) \ne 0$ in $\theta$. Then the iterative formula (28) given in Algorithm 1 produces a sequence of iterations $\{x_n:n=0,1,2,\ldots \}$ with order of convergence three.

#### Proof

Let

$$\begin{aligned} R(x) = x - \frac{f(x)}{f'(x)}-\frac{(f(x))^2f''(x)}{2(f'(x))^3}. \end{aligned}$$

Since $\alpha$ is a root of *f*(*x*), hence $f(\alpha ) = 0$. One can compute that

$$\begin{aligned}R(\alpha ) = \alpha , \\R'(\alpha ) = 0, \\R''(\alpha ) = 0, \\R'''(\alpha ) \ne 0. \end{aligned}$$

Hence the Algorithm 1 has third order convergence, by Theorem 1. $\square$

One can also verify that the order of convergence of Algorithm 1 as in the following example.

#### Example 1

Consider the following equation. It has a root $\alpha =\sqrt{30}$. We show, as discussed in proof of Theorem 2, that the Algorithm 1 has third order convergence.

31 $$\begin{aligned} ~ f(x) = 30-x^2. \end{aligned}$$

Following Theorem 2, we have

$$\begin{aligned} R(x) =~ x - \frac{f(x)}{f'(x)}-\frac{(f(x))^2f''(x)}{2(f'(x))^3} \\ =~ \frac{3x^4+180x^2-900}{8x^3}, \\ R'(x) =~ \frac{3 (x^2-30)^2}{8x^4}, \\ R''(x) =~ \frac{45 (x^2-30)}{x^5}, \\ R'''(x) =~ -\frac{135 (x^2-50)}{x^6}. \end{aligned}$$

Now

$$\begin{aligned} R(\alpha ) =~ \sqrt{30} = \alpha , \\ R'(\alpha ) =~ 0, \\ R''(\alpha ) =~ 0, \\ R'''(\alpha ) =~ \frac{1}{10} \ne 0. \end{aligned}$$

Hence, by Theorem 2, the Algorithm 1 has third order convergence.

#### Theorem 3

Let $f:I \rightarrow \mathbb {R}$. Suppose $\alpha \in I$ is a simple root of (1) and $\theta$ is a sufficiently small neighborhood of $\alpha$. Let $f''(x)$ exist and $f'(x) \ne 0$ in $\theta$. Then the iterative formula (29) given in Algorithm 2 produces a sequence of iterations $\{x_n:n=0,1,2,\ldots \}$ with order of convergence six.

#### Proof

Let

$$\begin{aligned} R(x) = x - \frac{f(x)}{f'(x)}-\frac{(f(x))^2f''(x)}{2(f'(x))^3} - \frac{f\left( x - \frac{f(x)}{f'(x)}-\frac{(f(x))^2f''(x)}{2(f'(x))^3} \right) }{f'\left( x - \frac{f(x)}{f'(x)}-\frac{(f(x))^2f''(x)}{2(f'(x))^3} \right) }. \end{aligned}$$

Since $\alpha$ is a root of *f*(*x*), hence $f(\alpha ) = 0$. One can compute that

$$\begin{aligned}R(\alpha ) = \alpha , \\R'(\alpha ) = 0, \\R''(\alpha ) = 0, \\R^{(3)}(\alpha ) = 0, \\R^{(4)}(\alpha ) = 0, \\R^{(5)}(\alpha ) = 0, \\R^{(6)}(\alpha ) \ne 0. \end{aligned}$$

Hence the Algorithm 2 has sixth order convergence, by Theorem 1. $\square$

We can also verify the order of convergence of Algorithm 2 as in the following example.

#### Example 2

Consider the equation (31). Using Theorem 3, similar to Example 1, we have

$$\begin{aligned} R(x) =~ x - \frac{f(x)}{f'(x)}-\frac{(f(x))^2f''(x)}{2(f'(x))^3} - \frac{f\left( x - \frac{f(x)}{f'(x)}-\frac{(f(x))^2f''(x)}{2(f'(x))^3} \right) }{f'\left( x - \frac{f(x)}{f'(x)}-\frac{(f(x))^2f''(x)}{2(f'(x))^3} \right) } \\ =~ \frac{3x^8+1000x^6+9000x^4-108000x^2+270000}{16x^3(x^4+60x^2-300)}, \\ R'(x) =~ \frac{(3x^8-280x^6+9000x^4-108000x^2+270000)(x^2-30)^2}{16x^4(x^4+60x^2-300)^2}, \\ R''(x) =~ \left( \frac{5(x^2-30)}{2x^5(x^4+60x^2-300)^3}\right) \\~~~~~~~(41x^{12}-3180x^{10}+60300x^8+684000x^6 \\~~~~~~-26730000x^4+145800000x^2-243000000), \\ R^{(3)}(x) =~ \left( -\frac{15}{2x^6(x^4+60x^2-300)^4}\right) \\~~~~~~(41x^{18}-9810x^{16}+488400x^{14}-3348000x^{12} \\~~~~~~-162900000x^{10}-955800000x^8+104868000000 x^6 \\~~~~~~-884520000000 x^4+2988900000000 x^2-3645000000000), \\ R^{(4)}(x) =~ \left( \frac{30}{x^7(x^4+60x^2-300)^5}\right) \\~~~~~~ (41x^{22}-17175x^{20}+1308000x^{18}-15727500x^{16} \\~~~~~~-296100000x^{14}-14625900000x^{12}+284580000000x^{10} \\~~~~~~+8460450000000x^8-117733500000000x^6 \\~~~~~~+650632500000000x^4-1662120000000000x^2 \\~~~~~~+1640250000000000), \\ R^{(5)}(x) =~\left( -\frac{150}{x^8(x^4+60x^2-300)^6}\right) \\~~~~~~(41x^{26}-26505x^{24}+3009600x^{22}-63693000x^{20} \\~~~~~~-95310000x^{18}-63030150000x^{16}-34344000000x^{14} \\~~~~~~+51666660000000x^{12}+470472300000000x^{10} \\~~~~~~-12040285500000000x^8+100252080000000000x^6 \\~~~~~~-407438100000000000x^4 +833247000000000000x^2 \\~~~~~~~-688905000000000000), \\ R^{(6)}(x) =~ \left( \frac{900}{x^9(x^4+60x^2-300)^7} \right) \\~~~~~~(41x^{30}-37800x^{28}+6113100x^{26}-208782000x^{24} \\~~~~~~+1884330000x^{22}-208202400000x^{20}-2671893000000x^{18} \\~~~~~~+144949500000000x^{16}+5848094700000000x^{14} \\~~~~~~+9219420000000000x^{12}-963361350000000000x^{10} \\~~~~~~+12133913400000000000x^8-71049069000000000000x^6 \\~~~~~~+227797920000000000000x^4-387755100000000000000x^2 \\~~~~~~+275562000000000000000). \end{aligned}$$

Now, we can check that

$$\begin{aligned}R(\alpha ) = \sqrt{30} = \alpha , \\R'(\alpha ) = 0, \\R''(\alpha ) = 0, \\R^{(3)}(\alpha ) = 0, \\R^{(4)}(\alpha ) = 0, \\R^{(5)}(\alpha ) = 0, \\R^{(6)}(\alpha ) = \frac{\sqrt{30}}{300} \ne 0. \end{aligned}$$

Hence, by Theorem 3, the Algorithm 2 has sixth order convergence.

#### Theorem 4

Let $f:I \rightarrow \mathbb {R}$. Suppose $\alpha \in I$ is a simple root of (1) and $\theta$ is a sufficiently small neighborhood of $\alpha$. Let $f''(x)$ exist and $f'(x) \ne 0$ in $\theta$. Then the iterative formula (30) given in Algorithm 3 produces a sequence of iterations $\{x_n:n=0,1,2,\ldots \}$ with order of convergence seven.

#### Proof

Let

$$\begin{aligned} R(x) =~ x - \frac{f(x)}{f'(x)}-\frac{(f(x))^2f''(x)}{2(f'(x))^3} - \frac{f\left( x - \frac{f(x)}{f'(x)}-\frac{(f(x))^2f''(x)}{2(f'(x))^3} \right) }{f'\left( x - \frac{f(x)}{f'(x)}-\frac{(f(x))^2f''(x)}{2(f'(x))^3} \right) } \\~~~-\frac{\left( f\left( x - \frac{f(x)}{f'(x)}-\frac{(f(x))^2f''(x)}{2(f'(x))^3} \right) \right) ^2 f''\left( x - \frac{f(x)}{f'(x)}-\frac{(f(x))^2f''(x)}{2(f'(x))^3} \right) }{2f'(x)\left( f'\left( x - \frac{f(x)}{f'(x)}-\frac{(f(x))^2f''(x)}{2(f'(x))^3} \right) \right) ^2}. \end{aligned}$$

Since $\alpha$ is a root of *f*(*x*), hence $f(\alpha ) = 0$. One can compute that

$$\begin{aligned}R(\alpha ) = \alpha , \\R'(\alpha ) = 0, \\R''(\alpha ) = 0, \\R^{(3)}(\alpha ) = 0,\\R^{(4)}(\alpha ) = 0, \\R^{(5)}(\alpha ) = 0, \\R^{(6)}(\alpha ) = 0, \\R^{(7)}(\alpha ) \ne 0. \end{aligned}$$

Hence the Algorithm 3 has seventh order convergence, by Theorem 1. $\square$

Again, one can verify the order of convergence of Algorithm 3 using the following example.

#### Example 3

Consider the equation (31). Following Theorem 4, similar to Example 1 and Example 2, we have

$$\begin{aligned} R(x) =~ x - \frac{f(x)}{f'(x)}-\frac{(f(x))^2f''(x)}{2(f'(x))^3} - \frac{f\left( x - \frac{f(x)}{f'(x)}-\frac{(f(x))^2f''(x)}{2(f'(x))^3} \right) }{f'\left( x - \frac{f(x)}{f'(x)}-\frac{(f(x))^2f''(x)}{2(f'(x))^3} \right) } \\~~~-\frac{\left( f\left( x - \frac{f(x)}{f'(x)}-\frac{(f(x))^2f''(x)}{2(f'(x))^3} \right) \right) ^2 f''\left( x - \frac{f(x)}{f'(x)}-\frac{(f(x))^2f''(x)}{2(f'(x))^3} \right) }{2f'(x)\left( f'\left( x - \frac{f(x)}{f'(x)}-\frac{(f(x))^2f''(x)}{2(f'(x))^3} \right) \right) ^2} \\ =~ \left( \frac{1}{512x^7(x^4+60x^2-300)^2} \right) (87x^{16}+39440x^{14}+2046800x^{12}\\~~~~~~+9912000x^{10} -428220000x^8+3650400000x^6-19116000000x^4 \\~~~~~~+58320000000x^2-72900000000). \end{aligned}$$

Now, we can check that

$$\begin{aligned}R(\alpha ) = \sqrt{30} = \alpha , \\R'(\alpha ) = 0, \\R''(\alpha ) = 0, \\R^{(3)}(\alpha ) = 0, \\R^{(4)}(\alpha ) = 0, \\R^{(5)}(\alpha ) = 0, \\R^{(6)}(\alpha ) = 0, \\R^{(7)}(\alpha ) = \frac{7}{300} \ne 0. \end{aligned}$$

Hence, by Theorem 4, the Algorithm 3 has seventh order convergence.

### Numerical example

This section presents several numerical examples to illustrate the proposed algorithms, and comparisons are made to confirm that the proposed algorithms give solution faster than existing methods.

#### Example 4

Consider a non-linear equation

32 $$\begin{aligned} x^2-e^x-3x+2=0. \end{aligned}$$

Suppose the initial approximation is $x_0 = 2$ with tolerance error $10^{-10}$ correct to ten decimal places. Following the proposed algorithms (in equations 28, 29 and 30), we have

$$\begin{aligned} x_0 =~2,\\ f(x) =~ x^2-e^x-3x+2~~\text {and}~~f(x_0) = -7.389056099, \\ f'(x) =~ 2x-e^x-3 ~~\text {and}~~f'(x_0) = -6.389056099,\\ f''(x) =2-e^x ~~\text {and}~~f''(x_0) =-5.389056099. \end{aligned}$$

Iteration-1 using Algorithm 1:

$$\begin{aligned} x_1 =~ x_0- \frac{f(x_0)}{f'(x_0)}-\frac{(f(x_0))^2f''(x_0)}{2(f'(x_0))^3} \\ =~ 0.2793895885. \end{aligned}$$

Iteration-2 using Algorithm 1:

$$\begin{aligned} x_1 =~0.2793895885,~~~~f(x_1) = -0.082432628, \\ f'(x_1) =~ -3.763543228, ~~f''(x_1) = 0.677677595. \end{aligned}$$

Now,

$$\begin{aligned} x_2 =~ x_1- \frac{f(x_1)}{f'(x_1)}-\frac{(f(x_1))^2f''(x_1)}{2(f'(x_1))^3} \\ =~ 0.2575298491. \end{aligned}$$

Iteration-3 using Algorithm 1:

$$\begin{aligned} x_2 =~0.2575298491,~~~~f(x_2) = 0.000001649, \\ f'(x_2) =~ -3.778670729, ~~~~f''(x_2) = 0.706269573. \end{aligned}$$

Now,

$$\begin{aligned} x_3 =~ x_2- \frac{f(x_2)}{f'(x_2)}-\frac{(f(x_2))^2f''(x_2)}{2(f'(x_2))^3} \\ =~ 0.2575302855. \end{aligned}$$

Similarly, the Iteration-4 using Algorithm 1 is $x_4 = 0.2575302855$. One can observe that Iteration-3 and Iteration-4 are same up to ten decimal places and also the tolerance error is $10^{-10}$. Hence the required approximate root of the given equation (32) is 0.2575302855.

Now, we compute the iterations using Algorithm 2 as follows.

Iteration-1 using Algorithm 2:

$$\begin{aligned} y_0= x_0- \frac{f(x_0)}{f'(x_0)}-\frac{(f(x_0))^2f''(x_0)}{2(f'(x_0))^3} = 0.2793895885, \\ x_{1}= x_0 - \frac{f(x_0)}{f'(x_0)}-\frac{(f(x_0))^2f''(x_0)}{2(f'(x_0))^3} - \frac{f(y_0)}{f'(y_0)} = 0.2574866574. \end{aligned}$$

Iteration-2 using Algorithm 2:

$$\begin{aligned} y_1= x_1- \frac{f(x_1)}{f'(x_1)}-\frac{(f(x_1))^2f''(x_1)}{2(f'(x_1))^3} = 0.2575302856, \\ x_{2}= x_1 - \frac{f(x_1)}{f'(x_1)}-\frac{(f(x_1))^2f''(x_1)}{2(f'(x_1))^3} - \frac{f(y_1)}{f'(y_1)} = 0.2575302853. \end{aligned}$$

Similarly, the Iteration-3 using Algorithm 2 is $x_3 = 0.2575302853$. One can observe that Iteration-2 and Iteration-3 are same up to ten decimal places and also the tolerance error is $10^{-10}$.

Now, the iterations using Algorithm 3 are as follows.

Iteration-1 using Algorithm 3:

$$\begin{aligned} y_0= x_0- \frac{f(x_0)}{f'(x_0)}-\frac{(f(x_0))^2f''(x_0)}{2(f'(x_0))^3} = 0.2793895885, \\ x_{1}= x_0 - \frac{f(x_0)}{f'(x_0)}-\frac{(f(x_0))^2f''(x_0)}{2(f'(x_0))^3} - \frac{f(y_0)}{f'(y_0)} -\frac{f(y_0)}{f'(y_0)} - \frac{(f(y_0))^2f''(y_0)}{2f'(x_0)(f'(y_0))^2} \\= 0.2574612148. \end{aligned}$$

Iteration-2 using Algorithm 3:

$$\begin{aligned} y_1= x_1- \frac{f(x_1)}{f'(x_1)}-\frac{(f(x_1))^2f''(x_1)}{2(f'(x_1))^3} = 0.2575302854, \\ x_{2}= x_1 - \frac{f(x_1)}{f'(x_1)}-\frac{(f(x_1))^2f''(x_1)}{2(f'(x_1))^3} - \frac{f(y_1)}{f'(y_1)} -\frac{f(y_1)}{f'(y_1)} - \frac{(f(y_1))^2f''(y_1)}{2f'(x_1)(f'(y_1))^2} \\= 0.2575302854. \end{aligned}$$

#### Example 5

Consider the following equations with corresponding initial approximations to compare results of the proposed three methods with other existing methods. We take tolerance error $10^{-15}$ with correct to 15 decimal places.

(*a*): $\cos x - x =0$ with initial approximation $x_0=1.7$,
(*b*): $xe^{-x} - 0.1 =0$ with initial approximation $x_0=0.1$,
(*c*): $\sin ^2 x - x^2 + 1 =0$ with initial approximation $x_0=-1$,
(*d*): $x-e^{\sin x}+1 =0$ with initial approximation $x_0=4$,
(*e*): $x^3-10 =0$ with initial approximation $x_0=1.5$.

Table 1 gives a comparison of iterations number with different methods. In the table, ER, NR, NM, A1, A2, A3 and DIV indicate Exact Root, Newton-Raphson method, Noor Method [2], Algorithms 1,  2 and  3 and diverges respectively.

**Table 1:** Comparing No. of iterations by different methods

Eq.	ER	NR	NM	A1	A2	A3
(a)	0.7390851332	5	3	3	2	2
(b)	0.1118325592	4	2	1	1	1
(c)	− 1.404491648	7	DIV	4	3	2
(d)	1.696812387	DIV	DIV	5	3	3
(e)	2.154434690	6	4	4	3	2

From Table 1, it is clear that the numerical results show that the proposed methods are more efficient than other existing methods.

### Mapleimplementation

In this section, we present implementation of the proposed Algorithms 1,  2 and  3 in Maple. Various maple implementations for differential and transcendental equations are available, see, for example [17–27, 35]. One can also implement the proposed algorithms in Microsoft Excel similar to the implementation of existing algorithms in [33, 34].

#### Pseudo code

**Input:** Given *f*(*x*); initial approximation *x*[0]; tolerance $\epsilon$; correct to decimal places $\delta$; maximum number of iterations *n*.

**Output:** Approximate solution

I. for *i* from 0 to *n* do
  II. Set $x[i+1]$ = formula (28), (29) or (30).
  III. if $|x[i+1]-x[i]| < \epsilon$ and $|f(x[i+1])| < \delta$ then break; Output $x[i+1]$

#### Maple code

We present the maple code of the proposed algorithms as follows, and sample computations presented in Section.

#### Algorithm 1 in Maple


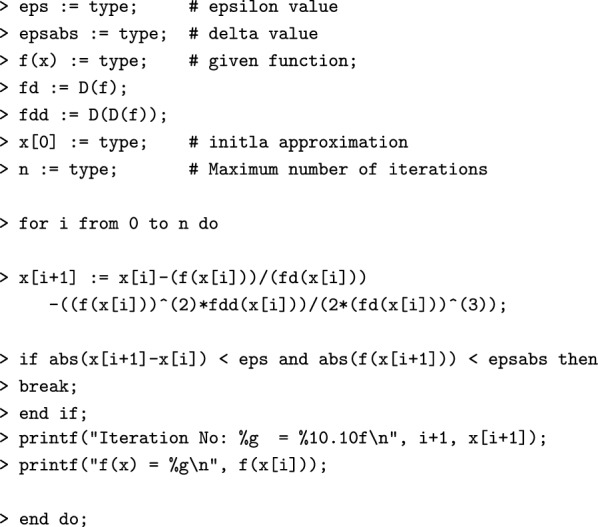


#### Algorithm 2 in Maple


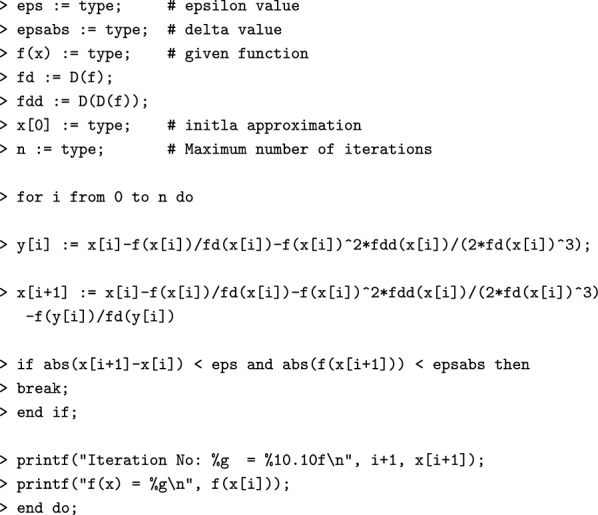


#### Algorithm 3 in Maple


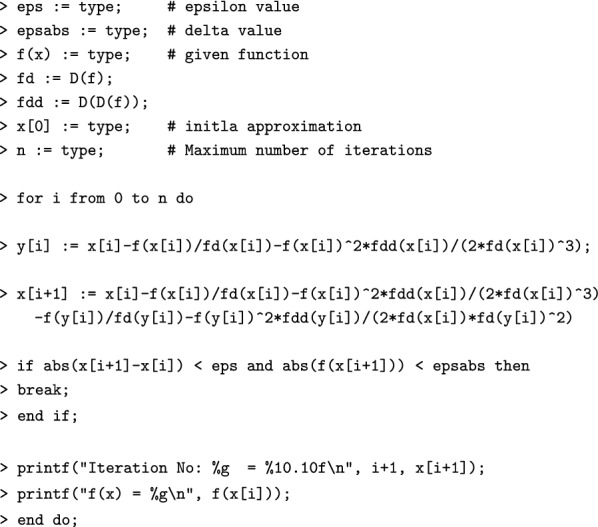


### Sample computations

Consider the following function for sample computations using the Maple implementation.

$$\begin{aligned} f(x) = \frac{1}{7} (30 - x^2), \end{aligned}$$

with initial approximation $x[0]=3.5$, tolerance $\epsilon = 10^{-5}$, correct to decimal places $\delta =10^{-10}$ (i.e., up to 10 decimal places);, and maximum number of iterations $n=10$.

#### Algorithm 1 sample computations using Maple


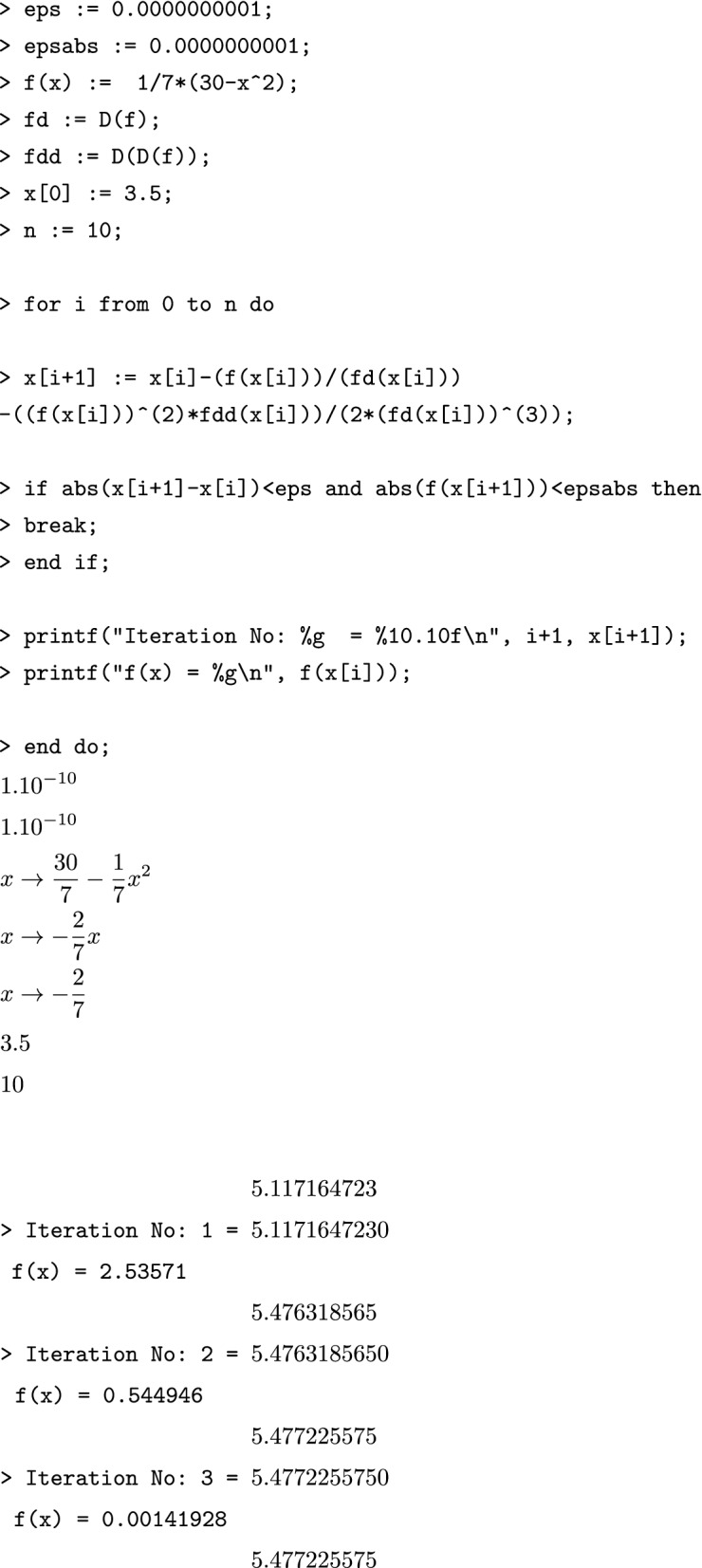

$$\begin{aligned}1.10^{-10} \\1.10^{-10} \\x \rightarrow \frac{30}{7} - \frac{1}{7}x^2 \\x \rightarrow - \frac{2}{7}x \\x \rightarrow - \frac{2}{7} \\3.5 \\10 \\5.117164723 \\ \texttt {> Iteration No: 1 = }5.1171647230 \\ \texttt {f(x) = 2.53571} ~~~~~~~~\\5.476318565 \\ \texttt {> Iteration No: 2 = }5.4763185650 \\ \texttt {f(x) = 0.544946} ~~~~~~\\5.477225575 \\ \texttt {> Iteration No: 3 = }5.4772255750 \\ \texttt {f(x) = 0.00141928} ~~~\\5.477225575 \end{aligned}$$

#### Algorithm 2 sample computations using Maple


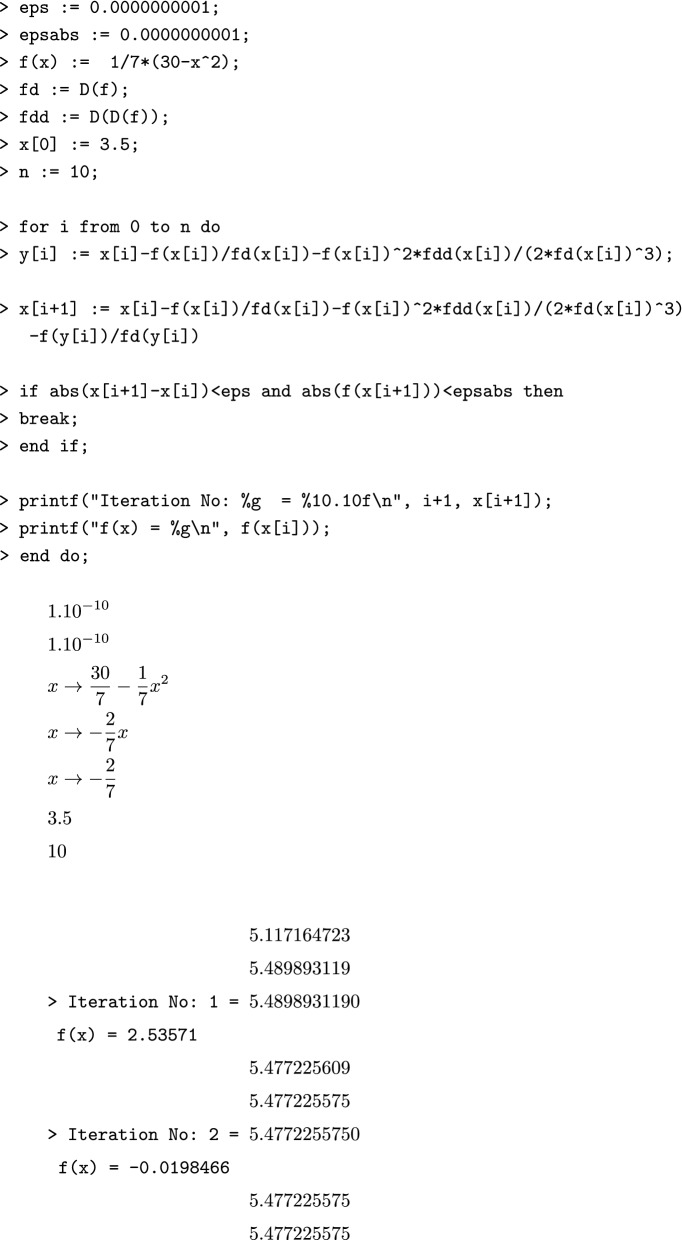

$$\begin{aligned}1.10^{-10} \\1.10^{-10} \\x \rightarrow \frac{30}{7} - \frac{1}{7}x^2 \\x \rightarrow - \frac{2}{7}x \\x \rightarrow - \frac{2}{7} \\3.5 \\10\\5.117164723 \\5.489893119 \\ \texttt {> Iteration No: 1 = }5.4898931190 \\ \texttt {f(x) = 2.53571} ~~~~~~~~\\5.477225609 \\5.477225575 \\ \texttt {> Iteration No: 2 = }5.4772255750 \\ \texttt {f(x) = -0.0198466} ~~~\\5.477225575 \\5.477225575 \end{aligned}$$

Similarly, one can apply the Algorithm 3 using Maple code.

### Conclusion

In this paper, we presented three iterative methods of order three, six and seven respectively for solving non-linear equations. With the help of modified homotopy perturbation technique, we obtained coupled system of equations which gives solution faster than existing methods. The analysis of convergence of the proposed iterative methods are discussed with example for each proposed method. Maple implementations of the proposed methods are discussed with sample sample computations. Numerical examples are presented to illustrate and validation of the proposed methods.

## Limitations

The proposed algorithms are implemented in Maple only. However, we can also implement these algorithms in Mathematica, SCILab, Matlab, Microsoft Excel etc.

## Data Availability

The datasets generated and analyzed during the current study are presented in this manuscript.

## Abbreviation

HPT: Homotopy Perturbation Technique
